# Draft Genome Sequences of Two Virulent Streptococcus equi subsp. *zooepidemicus* Swine Isolates from Pennsylvania

**DOI:** 10.1128/MRA.00974-20

**Published:** 2020-10-15

**Authors:** Meera Surendran Nair, Maurice Byukusenge, Lingling Li, Ruth H. Nissly, Victoria S. Cavener, Michele Yon, Rhiannon Barry, Pazhanivel Natesan, Nagaraja Thirumalapura, Deepanker Tewari, Bhushan M. Jayarao, Suresh V. Kuchipudi

**Affiliations:** a Animal Diagnostic Laboratory, Department of Veterinary and Biomedical Sciences, Pennsylvania State University, University Park, Pennsylvania, USA; b Department of Biology, Pennsylvania State University, University Park, Pennsylvania, USA; c Department of Pathology, Madras Veterinary College, Chennai, India; d Pennsylvania Veterinary Laboratory, Harrisburg, Pennsylvania, USA; e Center for Infectious Disease Dynamics, Pennsylvania State University, University Park, Pennsylvania, USA; University of Maryland School of Medicine

## Abstract

Draft genome sequences of two outbreak isolates of Streptococcus equi subsp. *zooepidemicus* from a Pennsylvania swine herd affected with high mortality and morbidity are reported here. The genome analysis revealed that the isolates are closely related to a virulent strain originally identified in China.

## Abstract (precis)

Draft genome sequences of two outbreak isolates of Streptococcus equi subsp. *zooepidemicus* from one of the Pennsylvania swine herds affected with high mortality and morbidity are reported here. The genome analysis revealed that the isolates are closely related to a virulent strain originally identified in China.

## ANNOUNCEMENT

Streptococcus equi subsp. *zooepidemicus* is a β-hemolytic, Gram-positive, Lancefield group C streptococcal bacterium. It is an opportunistic commensal in respiratory and reproductive tracts of horses (1, 2). Nevertheless, *S. equi* subsp. *zooepidemicus* is also known to infect multiple hosts, including cattle, sheep, pigs, camels, alpacas, foxes, birds, rabbits, guinea pigs, dogs, cats, and monkeys in addition to humans (2–7). In 2019, swine mortality events were reported in Ohio and Tennessee with clinical manifestations, including lethargy, weakness, and high fever, together with a swift spread of illness among pigs within the affected premises and rapidly escalating mortality levels (8). Similar clinical manifestations and severity of disease in swine were previously reported in China, and a genetic analysis revealed a virulent, invasive strain of *S. equi* subsp. *zooepidemicus* as the causative agent (9).

In late 2019, clinical samples from an affected swine herd with high mortality in Pennsylvania were submitted to the Pennsylvania Veterinary Laboratory (PVL). Two *S. equi* subsp. *zooepidemicus* isolates were cultured on blood agar for 48 h at 37°C, and identification was performed by matrix-assisted laser desorption ionization–time of flight (MALDI-TOF) mass spectrometry. DNA was extracted from an overnight 37°C-incubated brain heart infusion broth culture inoculated with single colonies using the PureLink genomic DNA minikit (Thermo Fisher Scientific, Waltham, MA). Illumina (San Diego, CA, USA) paired-end sequencing libraries were generated with the Nextera DNA Flex library prep kit (Illumina) and sequenced with the MiniSeq platform and midoutput reagent kit (2 × 150 bp). Base calling was performed with MiniSeq Local Run Manager software. *De novo* assembly of raw reads (total reads, 236,932 kb [isolate 1] and 299,687 kb [isolate 2]) was performed through the Unicycler v.0.4.4 pipeline using PATRIC v.3.6.5 (10), generating two draft genome assemblies, namely, A02199501 (64 contigs; *N*_50_, 57,599 bp; total size, 2.11 Mbp; GC content, 41.5%) and S1933003 (68 contigs; *N*_50_, 61,231 bp; total size, 2.11 Mbp; GC content, 41.54%). For all software used, default parameters were used unless otherwise indicated.

The assembled genomes were annotated with the NCBI Prokaryotic Genome Annotation Pipeline (PGAP v.4.11) (11). The A02199501 genome has 1,899 protein-coding sequences (CDSs), 28 tRNA genes, 3 rRNA genes, and 1 transfer-messenger RNA (tmRNA). Similarly, there are 1,897 CDSs, 28 tRNA genes, 3 rRNA genes, and 1 tmRNA in S1933003. Using BLAT analysis (12), high homology was observed between the two genomes among many known transporters, virulence factors, drug targets, and antibiotic resistance genes. The A02199501 and S1933003 genomes shared 99.4% similarity with respect to the CDSs. Genome analysis also revealed the presence of a streptococcal collagen-like protein-1 (Scl1) gene along with the antiphagocytic M-protein, indicating that isolates A02199501 and S1933003 are invasive and virulent like the *S. equi* subsp. *zooepidemicus* ATCC 35246 strain. Furthermore, A02199501 and S1933003 possess putative prophage integrases and prophage proteins in addition to a locus containing a clustered regularly interspaced short palindromic repeat (CRISPR) array.

### Data availability.

The draft genome sequences of the two Streptococcus equi subsp. *zooepidemicus* isolates have been deposited in GenBank under the accession numbers JABDID000000000 and JABMIH000000000. The raw reads have been submitted to the SRA database under the BioSample accession numbers SAMN14443823 and SAMN14443824.
